# Beyond Conventional MRI: Molecular PET, Multiparametric MRI, and PET/MRI in Glioma Management

**DOI:** 10.3390/diagnostics16213641

**Published:** 2026-11-04

**Authors:** Shu-Hang Ng, Tiing-Yee Siow, Hung-Wen Kao, Sheng-Chieh Chan, Sheng-Tzung Tsai

**Affiliations:** 1 Department of Medical Imaging and Intervention, Chang Gung Memorial Hospital at Linkou, College of Medicine, Chang Gung University, Taoyuan 333423, Taiwan; shuhangng@gmail.com (S.-H.N.); tiingyee@gmail.com (T.-Y.S.); 2 Department of Medical Imaging, Hualien Tzu Chi Hospital, Buddhist Tzu Chi Medical Foundation, Hualien 970423, Taiwan; cohimeow@gmail.com; 3 Department of Nuclear Medicine, Hualien Tzu Chi Hospital, Buddhist Tzu Chi Medical Foundation, Hualien 970423, Taiwan; 4 School of Medicine, College of Medicine, Tzu Chi University, Hualien 970423, Taiwan; 5 Department of Neurosurgery, Hualien Tzu Chi Hospital, Buddhist Tzu Chi Medical Foundation, Hualien 970423, Taiwan

**Keywords:** glioma, molecular imaging, positron emission tomography, magnetic resonance imaging, PET/MRI

## Abstract

Despite its central clinical role, conventional magnetic resonance imaging (MRI) provides an incomplete picture of the tumor biology of gliomas, which are biologically heterogeneous tumors. Molecular positron emission tomography (PET) with amino acid tracers, multiparametric MRI techniques (perfusion-, diffusion-, and spectroscopy-based sequences), and hybrid PET/MRI have each been proposed to overcome specific limitations of anatomic imaging in diagnosis, treatment planning, and posttreatment assessment. This narrative review examined the current evidence for molecular PET, multiparametric MRI, and integrated PET/MRI across the glioma care pathway. Rather than framing these modalities as competing alternatives, we argue that amino acid transport, vascularity, water diffusion, and metabolite composition capture distinct and often spatially incongruent facets of tumor biology, such that discordance between modalities may reflect the underlying tumor heterogeneity. We also summarized the guideline recommendations (RANO/EANO, EANM/SNMMI, etc.), outlined current evidence gaps, and discussed emerging directions in novel PET tracers, artificial intelligence, radiomics, and theranostics. Molecular PET and multiparametric MRI should be used as modalities complementary to conventional MRI within multidisciplinary glioma care, while the choice and sequence of examinations should be guided by the clinical question.

## 1. Introduction

Gliomas are the most common primary malignant brain tumors in adults. They comprise a heterogeneous group of diffuse central nervous system neoplasms characterized by distinct histologic and molecular features, including isocitrate dehydrogenase (IDH) mutation and 1p/19q-codeletion status [1]. The clinical relevance of these subtypes lies not only in the incidence, but also in infiltrative growth, neurologic morbidity, and recurrence despite multimodality treatment. This heterogeneity is central to the imaging problem in glioma, which extends beyond detecting a mass to estimating aggressiveness, defining tumor extent, selecting biopsy or resection targets, planning radiation, and separating viable tumor from the treatment effect.

Although conventional MRI remains indispensable for glioma diagnosis, treatment planning, and surveillance, it primarily depicts the anatomy, water content, mass effect, and blood–brain barrier disruption. However, enhancement does not necessarily imply a viable tumor, and a non-enhancing fluid-attenuated inversion recovery (FLAIR) abnormality may reflect tumor infiltration, edema, gliosis, or treatment-related change [2,3], driving interest in molecular PET, multiparametric MRI, and integrated PET/MRI.

Amino acid PET depicts the metabolically active tumor against a low-activity normal brain background, complementing MRI in the management of glioma [4,5,6]. Multiparametric MRI—perfusion-, diffusion-weighted, and spectroscopic imaging—adds physiologic and metabolic dimensions beyond conventional sequences [7], while hybrid PET/MRI integrates these signals spatially [8].

The central premise of this review is that molecular PET and multiparametric MRI are complementary, not competitive, sources of biologic information. Amino acid transport, glucose metabolism, vascularity, water diffusion, and metabolite composition each capture a different facet of tumor biology; discordance between modalities may signal spatial heterogeneity rather than error. This review covers molecular PET, multiparametric MRI, PET/MRI, guidelines, evidentiary gaps, and future directions, emphasizing actionable integration for nuclear medicine and neuro-oncology practice.

## 2. Literature Search and Selection

To identify relevant literature for this narrative review, we searched PubMed/MEDLINE on 31 August 2026 for articles published from 1 January 2005 through the date of the last search, using combinations of the terms “glioma,” “amino acid PET,” “[^18^F]FET,” “[^18^F]FDOPA,” “[^11^C]MET,” “[^18^F]fluciclovine,” “multiparametric MRI,” “perfusion,” “diffusion,” “MR spectroscopy,” and “PET/MRI.” English-language articles addressing glioma diagnosis, treatment planning, posttreatment assessment, and prognosis were considered, and the reference lists of the retrieved articles were screened for additional relevant publications. The titles and abstracts were screened by S.-C.C., and full texts were reviewed for studies considered relevant to each clinical question. When several studies addressed the same research question, guidelines and consensus statements and systematic reviews and meta-analyses were prioritized, followed by prospective or biopsy-validated studies and the largest retrospective cohort studies, respectively. The search was not intended to be exhaustive; article selection was guided by clinical relevance, with consideration of study design and methodological limitations. Moreover, because no formal risk-of-bias assessment was performed, the studies discussed should be regarded as representative of the available evidence.

## 3. Conventional MRI in Glioma Management: Essential Yet Limited Biologic Specificity

Conventional MRI constitutes the clinical foundation of glioma imaging. Standard protocols generally include pre- and postcontrast T1-weighted imaging, T2-weighted imaging, and FLAIR. Beyond their high spatial resolution and excellent soft tissue contrast, these sequences provide the anatomic reference basis for neuronavigation and radiotherapy target delineation. However, navigation based on preoperative MRI is not real-time, because its accuracy is limited by intraoperative brain shift; intraoperative MRI and ultrasound can provide updated information, and PET and other parametric maps could be coregistered and imported into neuronavigation systems. Furthermore, conventional MRI remains the imaging standard embedded in established response-assessment criteria [e.g., Response Assessment in Neuro-Oncology (RANO)] and therefore continues to guide treatment decisions and clinical trial endpoints.

Despite this pivotal role, the limitation of conventional MRI is that a single imaging appearance can correspond to several distinct biologic states. For example, T2/FLAIR hyperintensity may reflect gliosis, viable tumor, or treatment effect; similarly, contrast enhancement may represent either viable tumor tissue or merely a residual sign of posttreatment blood–brain barrier disruption. Conversely, certain biologically and clinically meaningful states may show no discernible imaging correlation at all. For instance, a viable tumor may show little or no contrast enhancement; similarly, infiltrative tumor cells may extend beyond the enhancing margin or present largely without enhancement altogether, as is often the case with lower-grade or IDH-mutant gliomas.

This mismatch becomes especially pronounced in the posttreatment setting. After surgery, new enhancement may reflect postoperative change, including enhancement around ischemic tissue at the resection margin. After radiotherapy or chemoradiotherapy, pseudoprogression and subsequent radiation necrosis can increase contrast enhancement and the T2/FLAIR signal, thereby mimicking tumor progression; similar inflammatory changes may occur under immunotherapy. Antiangiogenic therapy, by contrast, can elicit the opposite problem by normalizing abnormal vasculature and reducing permeability, such that contrast enhancement and the T2/FLAIR signal diminish or disappear even as the tumor continues to thrive or progress. Collectively, conventional MRI reliably depicts the anatomy but only indirectly reflects tumor biology and viability—a gap that has driven the growing use of complementary physiologic and molecular imaging.

## 4. Molecular PET for Biologic Characterization Beyond Conventional MRI

Molecular PET provides biologic information that is fundamentally different from the anatomic and structural information gleaned from conventional MRI. The most established PET agents for glioma are amino acid tracers, including O-(2-[^18^F]fluoroethyl)-L-tyrosine ([^18^F]FET), 3,4-dihydroxy-6-[^18^F]fluoro-L-phenylalanine ([^18^F]FDOPA), L-[methyl-11C]methionine ([^11^C]MET), and [^18^F]fluciclovine ([^18^F]FACBC). These tracers primarily exploit amino acid transport systems, especially large neutral amino acid transporters, which are upregulated in many gliomas (Table 1). Because physiologic amino acid uptake in normal cerebral cortex is generally lower than physiologic glucose uptake, amino acid PET often provides higher lesion-to-background contrast than [^18^F]FDG PET in many neuro-oncology scenarios [6].

Among the amino acid tracers, the evidence base for [^18^F]FET is among the most extensive, particularly from European studies. [^18^F]FET benefits from the practical advantages of fluorine-18 labeling, including a longer half-life that supports regional distribution. [^18^F]FDOPA has also been widely investigated and may be more accessible in some institutions or countries. The long history of [^11^C]MET in neuro-oncology renders it an important reference tracer in the literature, although its short carbon-11 half-life necessitates the provision of an on-site cyclotron. [^18^F]FACBC, originally developed and used clinically in other oncologic contexts, is another amino acid transport imaging agent, with growing evidence in glioma imaging and has recently garnered attention in Japanese practice guidance [2,6,9]. These tracers should not be regarded as interchangeable. They differ with respect to transport mechanisms, physiologic biodistribution, kinetic behavior, intracellular metabolism or retention, and practical availability. At present, no single amino acid tracer has been proven to be universally superior across all glioma imaging indications. Hence, in clinical practice, tracer selection is shaped not only by diagnostic performance but also by local regulatory approval, radiopharmaceutical availability, and institutional experience.

**Figure 1:**
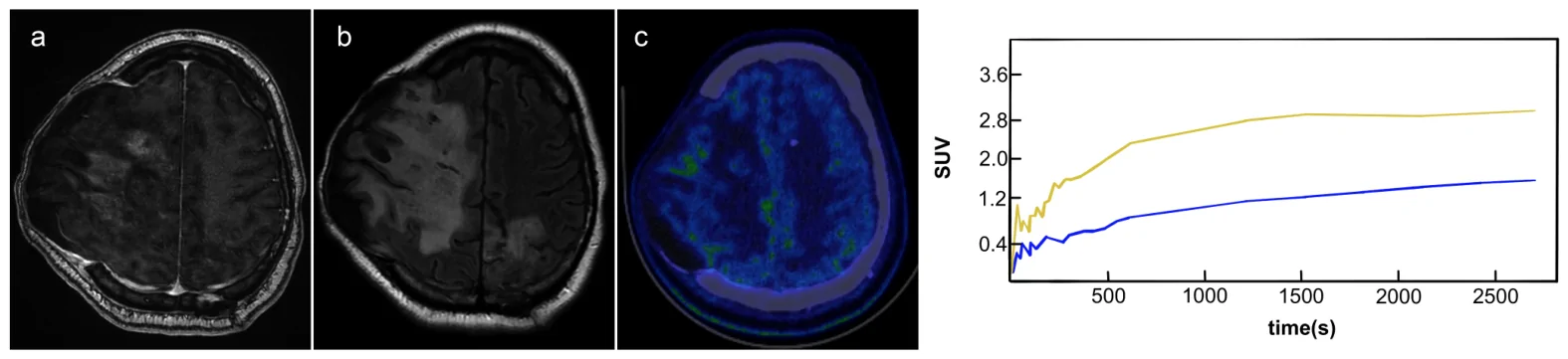
Utility of [^18^F]FET PET for evaluating MRI findings suspicious for a brain malignancy. A patient with a history of head trauma had persistent impairment of consciousness. Brain MRI demonstrated lesions suspicious for a neoplasm. (**a**) Axial contrast-enhanced T1-weighted image shows irregular patchy enhancement within the right frontal and parietal lobes. (**b**) Axial T2-weighted FLAIR image shows an extensive confluent hyperintense lesion involving the right frontal and parietal lobes. (**c**) The [^18^F]FET PET/CT image demonstrates mild tracer uptake in the corresponding region, and dynamic analysis of the lesion reveals a continuously increasing time–activity curve (yellow: lesion; blue: normal background brain). Taken together, these PET findings favor a non-neoplastic process. Histopathological examination of the right frontal biopsy specimen was consistent with progressive multifocal leukoencephalopathy with no evidence of tumor cells.

For the purpose of initial diagnosis and lesion characterization, amino acid PET can help distinguish neoplastic from non-neoplastic processes (Figure 1). This role is best understood as remedial rather than as replacement for MRI. [^18^F]FET PET has demonstrated high specificity in newly diagnosed cerebral lesions suspected to be glioma, supporting its use when conventional MRI cannot confidently separate the tumor from mimics [10,11,12]. Pooled evidence supports the diagnostic value of [^18^F]FET PET. A meta-analysis conducted by Dunet et al. reported a sensitivity and specificity of approximately 80% each for diagnosing primary brain tumors [11]. Nevertheless, amino acid uptake is not tumor-specific. Increased uptake may also be observed in metastases, lymphoma, meningioma, inflammatory or demyelinating lesions [13]. Therefore, amino acid PET should be interpreted in conjunction with MRI morphology and clinical context.

**Figure 2:**
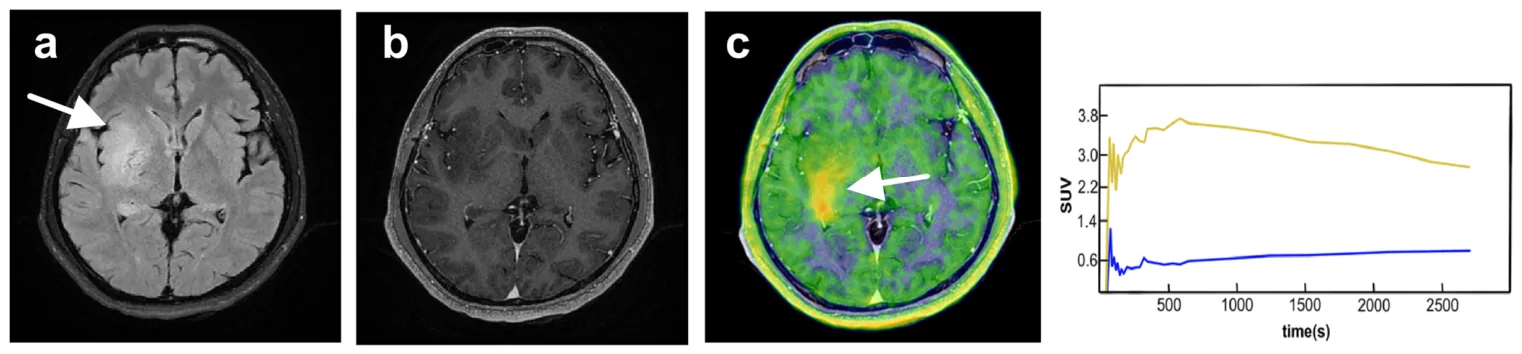
Utility of [^18^F]FET PET for detecting high-grade progression in a non-enhancing glioma. Follow-up MRI of a patient with a previously irradiated diffuse astrocytic glioma of the right insula (IDH-wildtype, NOS) with low-grade histologic features shows a non-enhancing lesion. (**a**) The axial T2-weighted FLAIR image demonstrates an extensive infiltrative hyperintense abnormality involving the right insula and basal ganglia (arrow). (**b**) The axial contrast-enhanced T1-weighted MRI image shows no conspicuous enhancement within the corresponding region. A recurrent non-enhancing glioma was suspected. (**c**) The corresponding fused [^18^F]FET PET/MRI image demonstrates marked tracer uptake in the right basal ganglia (arrow, mean TBR = 3.3). The time–activity curve of the lesion (yellow: lesion; blue: normal background brain) demonstrates an early peak followed by washout, a pattern suggestive of a higher-grade, metabolically active tumor. Histopathologic examination confirmed progression to glioblastoma, IDH-wildtype, CNS WHO grade 4. FLAIR: fluid-attenuated inversion recovery, MRI: magnetic resonance imaging, PET: positron emission tomography, CNS: central nervous system, WHO: World Health Organization, FET: fluoro-ethyl-tyrosine, TBR: tumor-to-background ratio.

Beyond simple lesion detection, molecular PET can provide information about tumor aggressiveness. On dynamic [^18^F]FET PET, higher-grade gliomas more frequently demonstrate an early uptake peak, followed by washout (Figure 2). In contrast, lower-grade tumors often show delayed or steadily increasing time–activity curves [3,10]. In newly diagnosed lower-grade glioma, these time–activity curve patterns and uptake parameters have been associated with high-risk tumor biology, showing promise for noninvasive prediction of the IDH-wildtype status [14,15]. Evidence from other amino acid tracers supports a similar biologic role: in a cohort of surgically treated lower-grade glioma, Ninatti et al. reported that a higher preoperative [^11^C]MET PET tumor-to-background ratio (TBR) independently predicted shorter progression-free survival in IDH-mutant astrocytomas [16].

Molecular PET can also guide biopsy targets and surgical planning by identifying metabolically active subregions within heterogeneous MRI abnormalities. Biopsy-validated analyses have shown that dynamic [^18^F]FET hotspots delineate malignant histopathologic features, supporting their use in selecting representative sampling sites [17]. Similarly, a prospective biopsy-validated study showed that [^18^F]FDOPA uptake identified high-grade tumor more frequently than contrast enhancement and extended beyond the enhancing margin, supporting its application in biopsy, resection, and radiotherapy planning [18]. The extent of residual [^18^F]FET-positive tumor was also associated with the outcome in a surgical series. Ort et al. found that gross-total resection of [^18^F]FET-defined biologic tumor volume, defined by a postoperative residual volume of <1 cm^3^, was associated with longer overall survival [19]. For [^18^F]fluciclovine, PET-positive but gadolinium-negative regions had a positive predictive value of 88.0% for the tumor, and PET findings changed the planned extent of resection in 47.2% of evaluable cases when MRI margins were uncertain [20]. Separately, Piroth et al. reported that a postoperative residual [^18^F]FET-avid volume of <25 mL was associated with longer median overall (20.0 vs. 6.9 months) and disease-free survival (9.6 vs. 5.1 months) than residual volume ≥ 25 mL (both *p* < 0.001) [21]. More recently, a bicentric retrospective study [22] reported that postoperative [^18^F]FET PET delineated residual tumor volumes substantially larger than those defined by MRI, with minimal spatial overlap between the two modalities across the RANO extent-of-resection classes [23]; moreover, residual PET volume was independently associated with overall survival, providing prognostic information beyond the MRI-derived tumor burden. Amino acid PET uptake and intraoperative 5-ALA fluorescence are related but not interchangeable. In a biopsy-controlled study of suspected gliomas, [^18^F]FET PET identified glioma tissue more frequently than 5-ALA fluorescence, particularly in lower-grade tissue, and all fluorescent samples were [^18^F]FET-positive [24]. In an IDH-wildtype glioblastoma series, [^18^F]FDOPA-positive regions beyond contrast enhancement showed tumor density similar to the enhancing core and corresponded to intense 5-ALA fluorescence [25].

Radiotherapy planning represents another major area in which molecular PET may complement MRI. In a prospective pattern-of-failure study, Harat et al. treated primary glioblastoma using MRI-defined targets, while pre-irradiation [^18^F]FET PET was used only for comparison. Recurrences more often involved the FET-defined than MRI-defined GTV (74% vs. 57%) [26]. Postoperative and pre-irradiation [^18^F]FET uptake as well as [^18^F]FET-derived biologic tumor volume have been associated with prognosis. Moreover, prospective treatment-planning studies have explored FET-adapted dose escalation [21,27,28,29].

**Figure 3:**
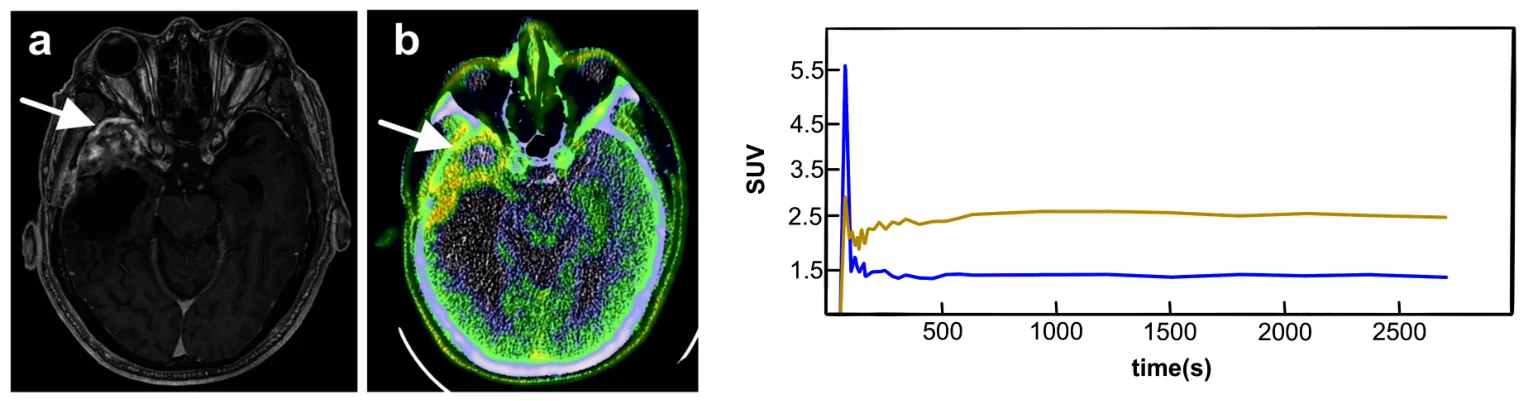
Utility of [^18^F]FET PET for differentiating treatment-related changes from recurrent glioblastoma. A patient with glioblastoma underwent follow-up imaging after surgical resection. (**a**) Axial contrast-enhanced T1-weighted MR image shows heterogeneous contrast enhancement at the anterior margin of the right temporal resection cavity, suspicious for recurrent disease (arrow). (**b**) Corresponding [^18^F]FET PET/CT scan shows only mild tracer uptake in the lesion (mean TBR = 1.03, arrow). On dynamic acquisition, the time–activity curve of the lesion reaches a plateau without washout (yellow: lesion; blue: normal background brain). Taken together, these PET findings favor a non-neoplastic process. Subsequent clinical and MRI follow-up confirmed treatment-related change without disease recurrence.

Molecular PET is also highly relevant in the posttreatment setting, particularly for differentiating tumor recurrence from treatment-related change (Table 2 and Figure 3). Galldiks et al. found that a static mean TBR ≥ 2.0 or a dynamic time-to-peak < 45 min increased the diagnostic accuracy for progressive or recurrent glioma from 75% with mean TBR alone to 93%, compared with 85% for conventional MRI [30]. Importantly, the usefulness of [^18^F]FET PET is not limited to the early pseudoprogression window. Kebir et al. reported that [^18^F]FET PET helped differentiate true progression from late pseudoprogression in glioblastoma, and Bashir et al. reported that 20-min static [^18^F]FET PET achieved 99% sensitivity and 94% specificity for detecting recurrent glioblastoma more than 6 months after radiotherapy [31,32]. A meta-analysis of [^18^F]FET parameters in primary and metastatic brain tumors reported a pooled sensitivity of 82% for static TBR, which increased to 90% when static and kinetic information were combined for differentiating true progression from treatment-related change [33].

Other PET tracers further broaden the evidence base for posttreatment glioma assessment. A meta-analysis reported favorable diagnostic performance for both [^18^F]FET and [^11^C]MET [34]. For [^18^F]FDOPA, static parameters identified recurrence or progression with at least 94% accuracy in a 51-patient cohort, while a subsequent 106-patient multicenter study found that at a threshold of 1.8, TBRmean differentiated progression from treatment-related change with 82% sensitivity and 71% specificity [35,36]. In [^18^F]fluciclovine PET, Ivanidze et al. found that the maximum standardized uptake value (SUVmax) was higher with true progression than with treatment-related change among 36 patients with presumed glioblastoma progression [37].

Molecular PET may also contribute to response assessment and prognostic stratification in the context of systemic or targeted therapies. In recurrent malignant glioma treated with bevacizumab-based therapy, the metabolic response on [^18^F]FET PET is associated with treatment failure and survival more consistently than changes in contrast enhancement in selected cohorts [38,39]. In the exploratory post hoc ARTE imaging analysis, persistent [^18^F]FET uptake on non-enhancing tumors predicted poorer survival (HR, 5.97), suggesting a metabolically active tumor, despite an apparent MRI response [40]. In a phase II cohort of patients treated with nivolumab and bevacizumab, [^18^F]FET PET-derived progressive volume on parametric response mapping after two treatment cycles distinguished responders with an area under the curve (AUC) of 0.843, whereas changes in contrast-enhancing MRI volume were not predictive [41]. [^18^F]FDOPA PET has likewise been investigated for antiangiogenic response assessment [42].

## 5. Multiparametric MRI Techniques: Physiologic and Metabolic Information Beyond Anatomy

Multiparametric MRI techniques extend the scope of conventional MRI by capturing physiologic and metabolic processes relevant to glioma biology. Perfusion-weighted imaging (PWI) characterizes hemodynamic and vascular properties, including perfusion, blood volume, and, depending on the technique, permeability. The most commonly used approaches include dynamic susceptibility contrast (DSC) PWI, dynamic contrast-enhanced (DCE) PWI, and arterial spin labeling (ASL). Diffusion-weighted imaging (DWI) and apparent diffusion coefficient (ADC) maps represent water mobility and tissue microstructure, serving as indirect markers of cellularity. MR spectroscopy (MRS) estimates metabolite concentrations or ratios involving choline (Cho), N-acetylaspartate (NAA), creatine, lactate, lipids, and, in selected protocols, the oncometabolite 2-hydroxyglutarate.

Perfusion MRI is the most widely used advanced sequence for assessing glioma vascularity, with relative cerebral blood volume (rCBV) supplementing conventional MRI in applications such as tumor grading and differentiation of recurrence from treatment-related change. A meta-analysis has shown favorable group-level diagnostic accuracy for the latter application [43], and rCBV remains a clinically informative adjunct to conventional MRI in individual cases. Perfusion MRI also helps distinguish glioblastoma from brain metastasis and primary CNS lymphoma, with high pooled diagnostic accuracy in meta-analyses [44,45]. However, the translation of rCBV into a single, cross-center actionable threshold is constrained by variability in acquisition, postprocessing, and reported optimal cutoffs, as well as inconsistent control for confounders such as antiangiogenic therapy, corticosteroid use, and histologic confirmation. ASL offers a non-contrast alternative, showing favorable group-level performance for glioma grading, although region-of-interest placement and acquisition and postprocessing methods varied considerably among the underlying studies [46].

DWI and its derivative metric ADC provide widely used, indirect surrogates for tissue cellularity. Unlike DSC and DCE perfusion imaging, DWI does not require contrast administration—a property it shares with ASL—and is included in virtually all standard clinical brain MRI protocols; meta-analyses report favorable group-level accuracy for both glioma grading and, in the posttreatment setting, differentiating recurrence from pseudoprogression [47,48]. DWI also contributes to distinguishing glioblastoma from primary CNS lymphoma [45], and a clinical practice guideline supports adding diffusion and perfusion imaging to anatomic MRI for this purpose in suspected glioblastoma [49]. However, as with rCBV, translating these estimates into a single validated cutoff remains challenging: reported thresholds for grading vary according to the field strength and b-value [47], and the recurrence-versus-pseudoprogression estimate is drawn from a comparatively small evidence base [48]. Diffusion tensor imaging (DTI) [50], which expands the scope of basic DWI to estimate the directionality of diffusion, enables the generation of white matter fiber orientation maps and fiber tractography, mapping white matter tract infiltration, displacement, or disruption, to guide maximal safe surgical resection. Diffusion kurtosis imaging (DKI) [51], another advanced DWI technique, assesses tissue microstructure by using higher b-values and more diffusion directions compared with DTI.

MRS measures metabolites such as Cho, NAA, creatine, lactate, and lipids. High-grade gliomas typically present elevated Cho levels, reflecting increased membrane turnover and cellular proliferation, along with reduced NAA, a marker of neuronal integrity [52]. Additional spectral peaks, such as lactate and lipid macromolecules, may appear in necrotic tumor cores. Beyond these individual metabolite changes, MRS can help delineate a metabolically abnormal tumor region beyond the margins visualized on structural MRI. However, MRS is constrained by long acquisition times, stemming from low metabolite concentrations, and by technical susceptibility to signal contamination from adjacent lipid or ventricular tissue and field inhomogeneity around surgical clips.

Newer methods such as APT-weighted CEST (APTw-CEST) imaging and MR fingerprinting may further expand the imaging biomarker landscape, but current evidence is preliminary and drawn from small, single-center proof-of-concept cohorts [53,54].

Collectively, multiparametric MRI provides complementary physiologic, microstructural, and metabolic information, though each technique reflects only an indirect correlate of tumor biology. Reported thresholds and diagnostic performance also vary across studies, confounded by largely indirect comparisons among techniques.

## 6. Molecular PET and Multiparametric MRI: Distinct Biologic Signals and Integrated Clinical Value

Direct comparisons between molecular PET and PWI are most informative when these modalities are viewed as mapping distinct, only partly overlapping aspects of glioma biology. Filss et al. found that [^18^F]FET PET delineated significantly larger tumor volumes than rCBV imaging (24.3 vs. 8.9 cm^3^; *p* < 0.001), with only 11% spatial congruence and a mean separation of 25.4 mm between their respective hotspots [55]. Similarly, Verger et al. demonstrated spatially incongruent FET and perfusion hotspots, supporting the interpretation that amino acid uptake and tumor perfusion reflect different pathophysiologic processes [56]. Thus, although metabolic activity and vascular proliferation may be related at the whole-tumor level, they are not interchangeable spatial markers of a biologically active tumor.

The clinical relevance of these modality-specific signals depends on the imaging question. In the prospective IMAGG study, [^18^F]FET PET-defined tumor volumes were significantly larger than contrast-enhanced MRI volumes (median, 43.5 vs. 23.8 mL; *p* = 0.005) and exhibited limited spatial similarity with contrast enhancement (median Dice coefficient, 0.52) [57]. In contrast, rCBV- and K2-defined abnormalities were largely contained within the contrast-enhancing volume, suggesting that [^18^F]FET PET may reveal metabolically active tumors beyond conventional MRI targets, whereas perfusion imaging may characterize vascular subregions within them. In the posttreatment setting, Panholzer et al. found that a three-parameter model combining [^18^F]FET TBRmean with DSC-MRI-derived rCBVmean and relative time to peak achieved higher diagnostic accuracy for differentiating progressive glioma from radiation necrosis than the best single parameter alone (AUC, 0.98 vs. 0.91 for TBRmean; sensitivity, 100% vs. 81%; *p* = 0.002) [58].

Comparisons between PET and MRS reveal a similar relationship. Using simultaneous hybrid PET/MRI, Mauler et al. found that [^18^F]FET-derived tumor volumes and regions with increased Cho/NAA were similar in average size but showed limited spatial agreement (mean Dice coefficient, 0.40) [59]. In a biopsy-validated study, Floeth et al. found that adding [^18^F]FET PET and single-voxel MRS to conventional MRI increased the accuracy in distinguishing neoplastic and non-neoplastic tissue from 68% to 97%, thereby improving diagnostic assessment and biopsy targeting [60].

**Figure 4:**
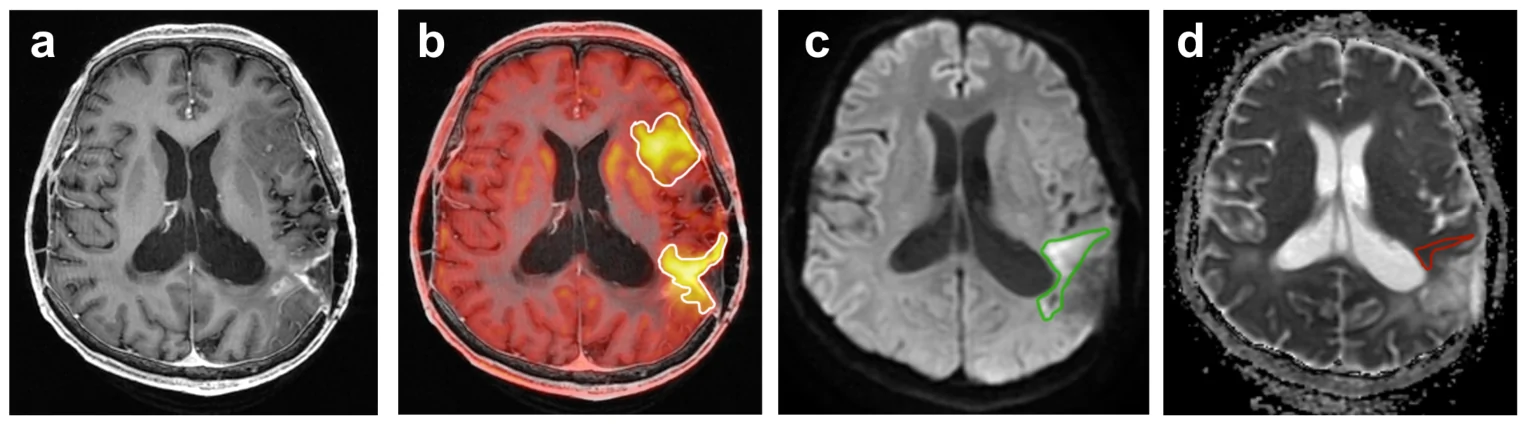
Limited spatial overlap between the [^18^F]FET-defined tumor regions and the diffusion-restricted region in recurrent glioblastoma. A patient with previously treated glioblastoma underwent follow-up imaging. (**a**) Axial contrast-enhanced T1-weighted MR image shows a small enhancing lesion medial to the resection cavity in the left parietal region, as well as a non-enhancing lesion in the left frontal lobe. (**b**) Fused [^18^F]FET PET/MRI image shows marked tracer uptake in the left frontal lesion and the left parietal lesion adjacent to the resection cavity (white contours). (**c**) The diffusion-weighted image shows a region of high signal intensity adjacent to the resection cavity (green contour), with corresponding low signal intensity on (**d**) the apparent diffusion coefficient (ADC) map (red contour), indicating restricted diffusion. The diffusion-restricted region is less extensive than, and only partially overlaps with, the regions of increased [^18^F]FET uptake. Subsequent MRI showed progression of both the left frontal and left parietal lesions, consistent with recurrent glioblastoma.

PET–DWI comparisons further illustrate both the potential and limitations of multimodal integration. Popp et al. found that diffusion-restricted regions showed minimal spatial agreement with FET-defined volumes (mean Dice coefficient, 0.10) in recurrent glioblastoma undergoing re-irradiation (Figure 4), and that 76% of the ADC-low volume was located outside regions of increased FET uptake [61]. To differentiate between treatment-related change and tumor progression, Werner et al. found that combining ADC with [^18^F]FET PET achieved higher diagnostic accuracy than ADC alone (89% vs. 69%) [62]. Cheng et al. conducted an exploratory PET/MR-guided biopsy study, reporting that combining [^18^F]FET uptake with ADC improved classification of high-grade glioma and selected molecular phenotypes, including human Telomerase Reverse Transcriptase (TERT)-mutant tumors, compared with [^18^F]FET PET alone (AUC, 0.850 vs. 0.711) [63]. Given the limited sample size and sample-level analysis, these molecular imaging findings require validation in larger cohorts.

Collectively, these studies show that molecular PET and multiparametric MRI provide biologically distinct yet clinically integrable information. Their combined use may improve specific tasks, including differentiating tumor progression from treatment-related changes, identifying biopsy targets, and refining treatment-volume delineation. However, the apparent spatial discordance between modalities does not necessarily reflect tumor heterogeneity. Registration errors, the lower spatial resolution of PET relative to MRI with associated partial-volume effects, differences in acquisition timing, and the thresholds used to define abnormal tissue on each modality can all affect the measured overlap; these technical factors should be considered before attributing discordant findings to biology.

## 7. Hybrid PET/MRI as a Technical Platform for Integrated Glioma Imaging

The principal technical advantage of hybrid PET/MRI is that it possesses the ability to compare spatially heterogeneous imaging abnormalities while minimizing registration errors and interval changes between examinations. Henriksen et al. demonstrated that although FET uptake and relative blood volume were quantitatively correlated, their spatial agreement was generally poor, with Dice coefficients exceeding 0.1 in fewer than half of positive cases [64]. Song et al. further showed that combining [^18^F]FET maximum TBR (TBRmax) with DSC-PWI-derived mean normalized cerebral blood volume (nCBVmean) improved differentiation of IDH-mutant astrocytoma from IDH-wildtype glioblastoma (AUC, 0.903 vs. 0.808 for TBRmax and 0.797 for nCBVmean alone) [65] (Table 3). Using anti-3-[^18^F]FACBC PET/MRI, Karlberg et al. found that adding PET information to MRI interpretation improved the prediction of glioma grade, tumor type, and IDH status, increasing the accuracy of the overall histomolecular diagnosis by 13.9–16.7 percentage points across two readers [66]. Bai et al. subsequently developed multiparametric [^18^F]FET PET/MRI radiomics models that outperformed individual modalities and an MRI-only model in an internal testing cohort, achieving AUCs of 0.97, 0.86, and 0.90 for predicting the IDH, TERT, and methylguanine-DNA methyltransferase (MGMT) status, respectively [67].

**Figure 5:**
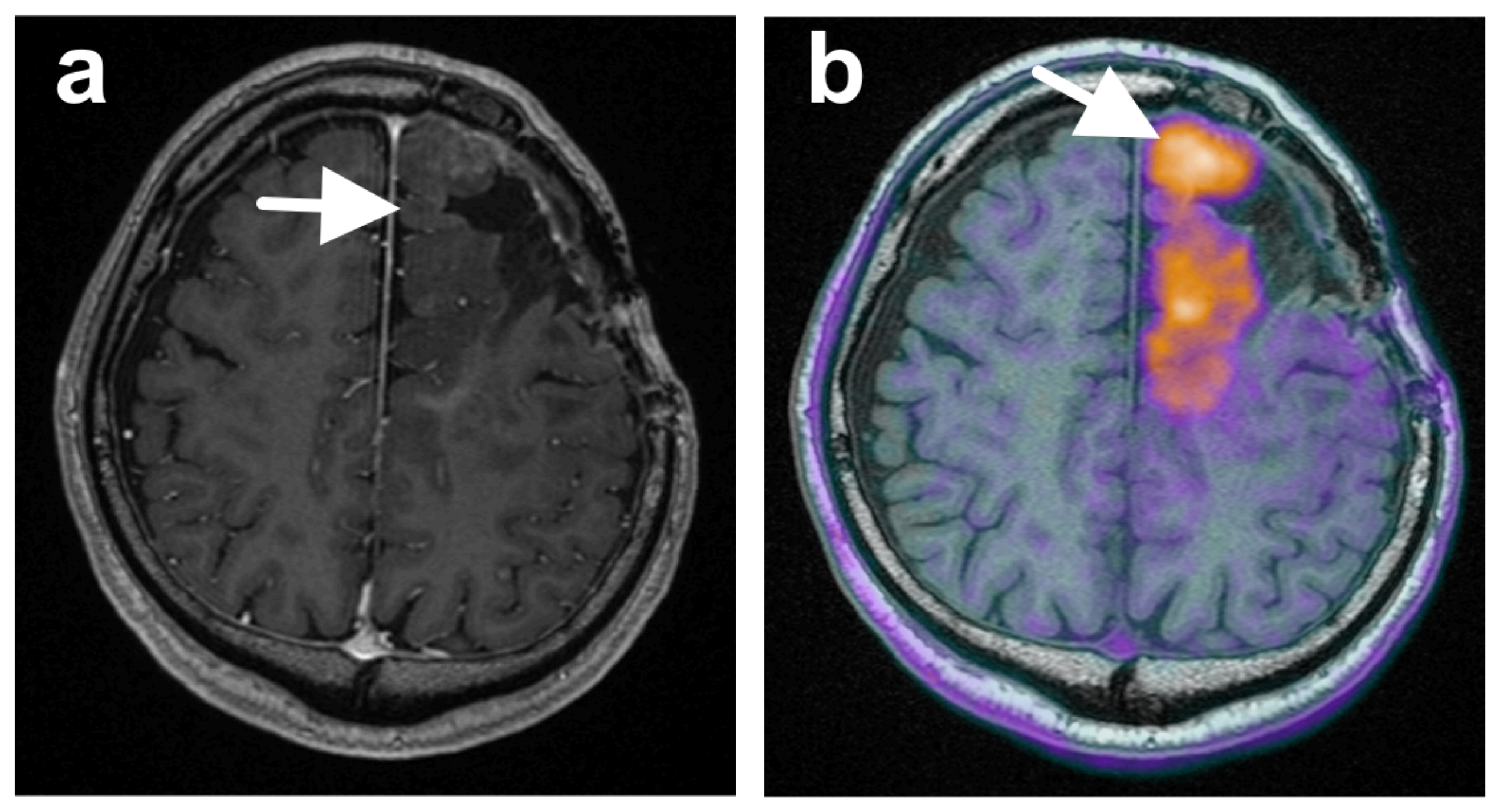
Illustrative case of [^18^F]FET PET/MRI-guided biopsy of recurrent glioma. A patient with a previously resected oligodendroglioma, IDH-mutant and 1p/19q-codeleted, CNS WHO grade 2, who had received adjuvant radiotherapy, developed a suspected recurrence in the left middle frontal lobe. (**a**) Postcontrast T1-weighted MRI shows only mild enhancement at the suspected lesion (arrow). (**b**) The fused [^18^F]FET PET/MRI image demonstrates a more extensive area of increased tracer uptake, with a focal hotspot selected for stereotactic biopsy (arrow). Histopathology confirmed recurrent oligodendroglioma, IDH-mutant and 1p/19q-codeleted, CNS WHO grade 3. This case illustrates how amino acid PET can identify metabolically active biopsy targets despite limited contrast enhancement. MRI: magnetic resonance imaging, PET: positron emission tomography, CNS: central nervous system, WHO: World Health Organization, FET: fluoro-ethyl-tyrosine.

Hybrid PET/MRI may also facilitate targeted biopsy and tumor delineation by pinpointing metabolically active tissue beyond conventional MRI abnormalities (Figure 5). Harat et al. conducted a prospective, serial biopsy study, finding that [^18^F]FET-avid regions extended beyond contrast enhancement and that including these regions in determining the biopsy target increased the final biopsy-determined tumor grade in 30% of cases [68]. Another biopsy-validated study by Song et al. reported that FET-defined tumor volumes were significantly larger than contrast-enhancing MRI volumes (77.8 vs. 34.6 cm^3^). All 21 biopsy samples obtained from FET-avid regions contained glioma tissue or tumor infiltration, whereas only 61.9% (13/21) of these sites showed MRI contrast enhancement [69]. Karlberg et al. reported that combined [^18^F]FACBC PET/MRI showed greater concurrence with malignant histopathology than contrast-enhanced MRI (89.5% vs. 26.3%) in image-localized biopsy analyses [70].

For distinguishing tumor progression from treatment-related change, hybrid PET/MRI facilitates direct evaluation of metabolic findings against diffusion and perfusion abnormalities. D’Amore et al. conducted a retrospective exploratory study by using [^18^F]FET-positive volumes to guide diffusion kurtosis imaging analysis. A model combining TBRmax with kurtosis metrics achieved an AUC of 0.97 and accuracy of 94% for differentiating true progression from treatment-related change [71]. Smith et al. evaluated hybrid [^18^F]FET perfusion PET/MRI in a heterogeneous cohort of 42 high-grade gliomas and 38 brain metastases with equivocal conventional MRI findings; accuracy in the glioma subgroup was 86%. When PET and perfusion MRI were discordant (15 of 80 patients, whole cohort), [^18^F]FET PET indicated the correct diagnosis in 12 (80%), whereas perfusion MRI was correct in 3 patients (20%). Clinical management was changed after 88% of diagnoses of disease progression [72].

The prognostic evidence remains exploratory. Henssen et al. reported that [^18^F]FET-derived biological tumor volume and the number of metabolically active lesions were associated with overall survival in recurrent high-grade glioma, whereas TBRmax, rCBVmax, and the Cho/NAA ratio were not [73].

However, evidence does not consistently show that combining PET and MRI parameters improves diagnostic performance beyond the stronger individual modality. Verger et al. found comparable grading performance for [^18^F]FET PET and perfusion-weighted imaging in patients with newly diagnosed, untreated gliomas, but combining PET with perfusion parameters did not further improve the diagnostic accuracy [56]. Similarly, Mauler et al.’s biopsy-controlled hybrid PET/MRI study of patients with suspected glioma found no additional benefit of combining [^18^F]FET PET with normalized Cho/NAA MR spectroscopic imaging beyond either modality alone [74].

Hybrid PET/MRI acquisition offers potential practical advantages, including the absence of an interval between examinations, which is particularly relevant for neuronavigation and biopsy planning; greater patient comfort; and the acquisition of multiparametric MRI during the PET scan without prolonging the examination [68]. However, to our knowledge, no prospective study has directly compared hybrid acquisition with separately acquired and coregistered PET and MRI. Therefore, it is unclear whether the diagnostic benefits reported in the above-mentioned hybrid PET/MRI studies derive from simultaneous acquisition itself or from the combination of PET and MRI information, which can also be obtained from separate examinations. Prospective comparative studies are needed to determine whether hybrid PET/MRI improves diagnostic performance beyond the combined interpretation of separately acquired PET and MRI, and whether any incremental benefit justifies its greater cost and workflow complexity.

## 8. Clinical Integration: Guidelines and Recommendations

Guidelines and consensus recommendations increasingly recognize amino acid PET as a complementary tool in glioma imaging. The initial 2016 RANO/European Association of Neuro-Oncology (EANO) recommendations summarized evidence for the role of PET in differential diagnosis, delineation of tumor extent, biopsy and resection guidance, radiotherapy planning, differentiation of recurrence from treatment-related changes, treatment response assessment, and prognostication, using an Oxford level-of-evidence framework, albeit without indication-specific recommendation strength categories [3]. The 2025 update substantially refines and broadens this framework by formally grading recommendations as strong, moderate, or weak. Evidence levels were upgraded from level 3 to level 2 for several established indications, including delineation of glioma extent, differentiation of recurrence from treatment-related changes, detection of malignant tumor components within non-enhancing gliomas, and response assessment; the first three received strong recommendations, whereas response assessment received a moderate recommendation [4]. The update also incorporates PET RANO 1.0 [75] for standardized response assessment and introduces a dedicated, albeit weak, recommendation for amino acid PET in pediatric patients with glioma.

For imaging acquisition and interpretation, the joint EANM/EANO/RANO practice guidelines and SNMMI procedure standards provide practical recommendations for patient preparation, acquisition, reconstruction, quantification, and reporting of amino acid and [^18^F]FDG PET in gliomas [76]. They also specify standardized acquisition time windows for each tracer, recommend coregistration with recent contrast-enhanced T1-weighted and FLAIR MRI, and summarize TBR thresholds according to the clinical question, together with common interpretive pitfalls. On the MRI side, the consensus Brain Tumor Imaging Protocol defines a minimum standardized set of MRI sequences for brain tumor imaging [77], and a compatible consensus protocol standardizes DSC perfusion acquisition and postprocessing to reduce variability in rCBV measurements [78]. RANO 2.0 provides unified MRI-based response criteria for adult gliomas and states that advanced MRI techniques and amino acid PET may help distinguish progression from pseudoprogression, albeit warranting further validation before formal incorporation [79].

In radiotherapy, the PET/RANO report on PET imaging for radiotherapy planning and monitoring, the ESTRO-EANO guideline on target delineation, and recent practical guidance on [^18^F]FET PET-based target-volume delineation collectively support PET as an adjunct in selected planning scenarios [80,81,82]. These recommendations do not imply that PET is a replacement for MRI, histopathology, or clinical judgment; rather, they define areas where molecular imaging may mitigate uncertainty and improve consistency.

## 9. Current Limitations and Evidence Gaps

Several limitations constrain the routine use of molecular PET, multiparametric MRI, and PET/MRI. Tracer availability remains inconsistent across regions. [^11^C]MET requires an on-site cyclotron, whereas [^18^F]FET, [^18^F]FDOPA, and [^18^F]fluciclovine may be more suitable for regional distribution but are beset by variable approval, reimbursement, and integration into clinical workflows. Protocol differences in acquisition duration, reconstruction, tumor-to-background measurement, and threshold selection can influence the results of quantitative PET. A further limitation of amino acid PET is that a negative scan does not exclude the possibility of glioma. Approximately 20–30% of IDH-mutant CNS WHO grade 2 gliomas do not show increased amino acid uptake [6], and the joint procedure guidelines likewise caution that low uptake does not exclude a lower-grade astrocytoma [76].

Multiparametric MRI has parallel limitations. PWI depends on the acquisition technique, arterial input assumptions, and post-processing choices. DWI and ADC are influenced by edema, necrosis, hemorrhage, and scanner-related factors. MRS requires technical expertise, careful voxel placement or volumetric acquisition, and quality control. Multiparametric imaging can improve confidence, but can also create interpretive complexity in the event of parameter disagreement.

The reference standard used in diagnostic studies may limit the strength of the evidence. Histopathology is itself an imperfect reference for heterogeneous gliomas, because biopsy samples only a small part of the lesion and may miss the most aggressive component or the region corresponding to the imaging abnormality. In the posttreatment setting, histologic confirmation is usually available for only a subset of patients, and the remainder are classified by clinical and radiological follow-up. Such mixed reference standards can introduce verification bias and may inflate or distort the reported diagnostic accuracy, necessitating caution during interpretation.

Nevertheless, the gaps in evidence are substantial. Many studies employed a retrospective design, a single-center setting, or included modest sample sizes. Clinically meaningful endpoints such as management change and quality of life have been assessed in few studies, and data on cost-effectiveness are scarce.

## 10. Practical Integration by Clinical Question

**Figure 6:**
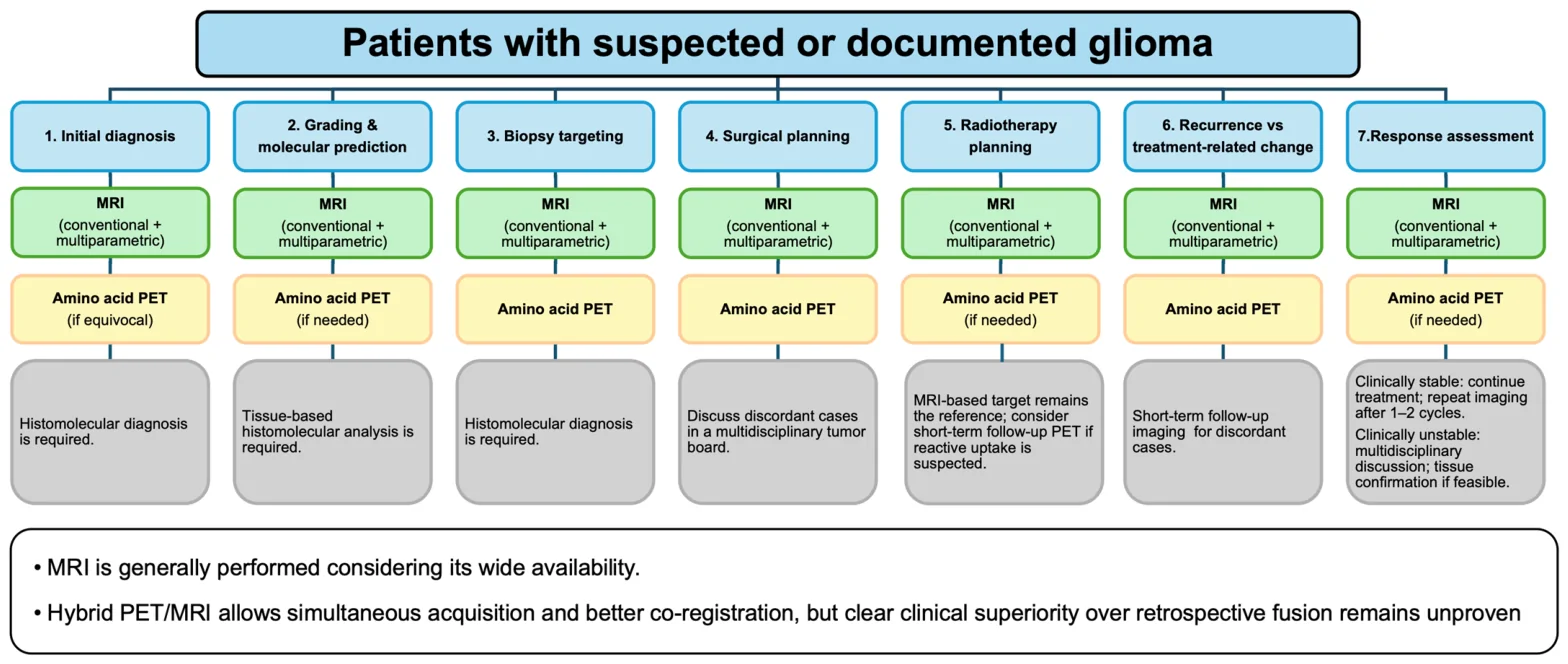
Schematic overview of imaging integration by clinical question in glioma. For each clinical question, MRI is generally performed first, and amino acid PET is added when the question remains unresolved and PET is accessible. The bottom row (gray) summarizes what may be considered when MRI and PET findings remain inconclusive or discordant. Supporting references are given in Table 4. MRI: magnetic resonance imaging, PET: positron emission tomography.

The information that conventional and multiparametric MRI and amino acid PET can provide for common clinical questions, and what may be considered when findings remain inconclusive or discordant, is summarized in Table 4 and Figure 6. Because MRI is widely available and relatively inexpensive, it is generally performed first; amino acid PET is added when a specific question remains unresolved after MRI, and PET is accessible, consistent with the updated RANO/EANO recommendations [4].

When PET and MRI findings are discordant, technical factors, including registration error, differences in spatial resolution, acquisition timing, and the thresholds used to define abnormal tissue, should be considered first. Thereafter, the approach depends on the clinical question. In newly diagnosed lesions, absent amino acid uptake does not exclude glioma, and histomolecular diagnosis remains decisive for diagnosis, grading, and biopsy targeting [4]. For resection planning, PET-avid regions beyond contrast enhancement may contain tumor tissue [68,69,80], and the extent of resection is weighed against the risk of neurological deficit during multidisciplinary discussion. For radiotherapy planning, the MRI-based target remains the reference, and short-term follow-up PET before irradiation can be considered when reactive postoperative uptake is suspected [81,82]. In suspected recurrence, short-term follow-up imaging is the usual approach, with confirmatory MRI recommended when progression is suspected within 12 weeks after radiotherapy [75,79]. During systemic therapy, clinically stable patients with equivocal findings may continue treatment under close observation with repeat imaging [75,79]. Multidisciplinary discussion and, where feasible, tissue confirmation should be considered in clinically unstable patients.

## 11. Emerging Research Directions

Novel PET tracers may broaden the information available beyond amino acid transport. Fibroblast activation protein inhibitor (FAPI) PET is one emerging example: tissue-based and imaging data suggest that FAPI uptake correlates with fibroblast activation protein expression in glioblastoma and gliosarcoma, and prospective [^68^Ga]FAPI-46 data support its feasibility in glioma visualization and recurrence assessment [85,86,87].

Artificial intelligence is also poised to expand the value of molecular imaging via synthetic PET generation and multiparametric prediction models. MRI-based prediction and synthesis of [^18^F]FET PET information is one such application under investigation [88]. Radiomics, which extracts quantitative features directly from acquired PET data, offers a complementary approach: static [^18^F]FET PET radiomics has demonstrated added diagnostic value over conventional TBR-based parameters for differentiating treatment-related changes from tumor progression in glioma [89]. Radiomics features reflecting tumor heterogeneity have further emerged as independent prognostic markers for progression-free and overall survival in glioblastoma after chemoradiation [90].

Theranostic strategies represent another emerging direction, but their role in glioma remains highly exploratory. Prostate-specific membrane antigen (PSMA) expression has been described in the tumor-associated neovasculature of glioblastoma, providing a rationale for PSMA-targeted imaging or therapy in selected high-grade gliomas [91]. Early clinical reports of [^68^Ga]PSMA imaging and [^177^Lu]PSMA therapy for recurrent glioblastoma or high-grade glioma suggest technical feasibility, but the evidence is limited to proof-of-concept reports, small experiences, and dosimetry-focused statements [92,93]. Accordingly, glioma theranostics should be considered conservatively, contingent upon further prospective validation.

## 12. Conclusions

Conventional MRI remains the essential foundation of glioma imaging. Amino acid PET and diffusion, perfusion, and spectroscopic MRI each provide an indirect and partial view of tumor biology—transport activity, microstructure, vascularity, and metabolite composition, respectively. Therefore, the most useful clinical framework is not PET versus MRI but question-driven integration: molecular PET may help when MRI findings are indeterminate, when biopsy or treatment planning requires biologically representative targeting, or when the distinction between recurrence and treatment-related change is ambiguous, while PET/MRI may further refine this assessment by mapping vascular, cellular, and metabolic heterogeneity within the same tumor.

Realizing this potential will require harmonized acquisition protocols and prospective multicenter validation, along with careful integration of novel tracers, artificial intelligence, radiomics, and cautiously framed theranostic concepts.

## Figures and Tables

**Table 1:** PET tracers relevant to glioma.

Tracer	Biologic Signal	Main Advantage	Key Limitation
[^18^F]FET	Amino acid transport	Strong European evidence; dynamic parameters	Variable access in some regions
[^18^F]FDOPA	Amino acid transport and decarboxylation	Fluorine-18 logistics; used in several regions	Physiologic basal ganglia uptake
[^11^C]MET	Amino acid transport/protein synthesis-related signal	Long neuro-oncology history	On-site cyclotron required
[^18^F]Fluciclovine/FACBC	Amino acid transport	Regional relevance in Japan	Evidence base smaller than [^18^F]FET/FDOPA/MET
FAPI/PSMA tracers	Tumor microenvironment/neovasculature targets	Potential non-amino-acid biology	Investigational; limited glioma outcome data

PET: positron emission tomography; [^18^F]FET: O-(2-[^18^F]fluoroethyl)-L-tyrosine; [^18^F]FDOPA: 3,4-dihydroxy-6-[^18^F]fluoro-L-phenylalanine; [^11^C]MET: L-[methyl-11C]methionine; [^18^F]FACBC: [^18^F]fluciclovine; FAPI: fibroblast activation protein inhibitor; PSMA: prostate-specific membrane antigen.

**Table 2:** Molecular PET diagnostic performance for recurrence versus treatment-related change.

Study/Tracer	Study Design	Reference Standard	N	Sensitivity (95% CI)	Specificity (95% CI)	Accuracy/AUC (95% CI)
Galldiks et al. [^18^F]FET [30]	Single-center retrospective	Histopathology or Follow-up	124 patients	93%	100%	93%
Kebir et al. [^18^F]FET [31]	Single-center retrospective	Follow-up	26 patients	84%	86%	85%; AUC 0.88 (0.73–1.0)
Bashir et al. [^18^F]FET [32]	Single-center retrospective	Histopathology or Follow-up	146 patients	99%	94%	99%; AUC 0.97
Diao et al. [^18^F]FET [33] *	Meta-analysis	Histopathology or Follow-up	17 studies	Static 82% (78–85); Static + kinetic 90% (84–94)	Static 84% (79–89); Static + kinetic 90% (80–96)	--
de Zwart et al. [34]	Meta-analysis	Histopathology or Follow-up	39 studies/11 tracers	FET 90% (81–95); MET 93% (80–98); FDG 84% (72–92)	FET 85% (71–93); MET 82% (68–91); FDG 84% (69–93)	--
Zaragori et al. [^18^F]FDOPA [35]	Single-center retrospective	Histopathology or Follow-up	51 patients	94%	94%	94%; AUC 0.983 (0.96–1.0)
Rozenblum et al. [^18^F]FDOPA [36]	Multicenter retrospective	Histopathology or Follow-up	106 patients	82%	71%	78%; AUC 0.79 (0.69–0.89)
Ivanidze et al. [^18^F]fluciclovine [37]	Multicenter retrospective	Histopathology or Follow-up	36 patients	86% (70–99)	86% (64–99)	AUC 0.90 (0.79–0.98)

PET: positron emission tomography; [^18^F]FET: O-(2-[^18^F]fluoroethyl)-L-tyrosine; [^18^F]FDOPA: 3,4-dihydroxy-6-[^18^F]fluoro-L-phenylalanine; FDG: fluorodeoxyglucose; AUC: area under the curve; CI: confidence interval. * Gliomas (13 studies) and brain metastases (4 studies) pooled. CIs are shown where reported in the original publications.

**Table 3:** Representative PET/MRI cohort studies.

Study	N	Clinical Setting	PET/MRI Components	Main Message	Reference Standard
Verger et al. [56]	72	Preoperative glioma grading	[^18^F]FET PET + DSC-PWI	Both modalities contributed to grading information, with frequent spatial incongruence	Histopathology
Song et al. [65]	52	Preoperative histomolecular classification	[^18^F]FET PET + DSC-PWI	TBRmax plus nCBVmean improved selected IDH-related classification	Histopathology
Karlberg et al. [66]	36	Preoperative diagnostic and histomolecular characterization	[^18^F]FACBC PET + multiparametric MRI	PET added to MRI improved predicted grade, type, IDH status, and overall diagnosis	Histopathology
Bai et al. [67]	97	Preoperative molecular- genotype prediction	Multiparametric [^18^F]FET PET/MRI radiomics	Multimodal radiomics predicted IDH, TERT, and MGMT status	Histopathology
Mauler et al. [59]	41	Tumor delineation and spatial characterization	[^18^F]FET PET + volumetric MRS	[^18^F]FET-derived tumor volume and Cho/NAA-defined metabolic abnormality are spatially distinct	Not applicable
Harat et al. [68]	23	Biopsy targeting and malignant- region localization	[^18^F]FET PET + contrast-enhanced MRI/FLAIR + serial biopsy	Adding amino acid PET to MRI improved localization of malignant regions	Histopathology
Song et al. [69]	33	Biopsy targeting and tumor delineation	[^18^F]FET PET + contrast-enhanced MRI	Hybrid PET/MR improved delineation of tumor spatial biodistribution against biopsy validation	Biopsy histopathology (7 of 33 patients)
Karlberg et al. [70]	11	Diagnostic assessment and neurosurgical planning	[^18^F]FACBC PET + conventional MRI	Improved lesion detection and biopsy concordance compared with contrast-enhanced MRI	Histopathology; localized biopsies (5 of 11 patients)
D’Amore et al. [71]	32	Posttreatment progression assessment	[^18^F]FET PET + PET-guided DKI	TBRmax plus kurtosis metrics differentiated true progression from treatment-related change	Histopathology or follow-up
Smith et al. [72]	80	Posttreatment progression assessment	[^18^F]FET PET + DSC-PWI and DCE-PWI	Integrated interpretation may help resolve difficult discordant cases	Histopathology or follow-up
Henssen et al. [73]	26	Prognostic assessment in recurrent disease	[^18^F]FET PET + DSC-PWI and MRS	Biological tumor volume predicted survival	Not applicable

DCE-PWI: dynamic contrast-enhanced perfusion-weighted imaging; DWI: diffusion-weighted imaging; MRS: MR spectroscopy; DKI: diffusion kurtosis imaging; [^18^F]FET: O-(2-[^18^F]fluoroethyl)-L-tyrosine; [^18^F]FACBC: [^18^F]fluciclovine; TBRmax: maximum tumor-to-background ratio; nCBVmean: mean normalized cerebral blood volume; IDH: isocitrate dehydrogenase; Cho: choline; NAA: N-acetylaspartate; TERT: Telomerase Reverse Transcriptase; MGMT: methylguanine-DNA methyltransferase. Reference standard: histopathology refers to patient-level diagnosis; biopsy histopathology refers to image-guided samples compared with imaging findings at the sampled sites. Smith et al. [72] included 42 gliomas and 38 brain metastases.

**Table 4:** Summary of the information provided by MRI and amino acid PET for common clinical questions in glioma.

Clinical Question	Conventional and Multiparametric MRI	Amino Acid PET	If MRI and PET Are Inconclusive or Discordant
Initial diagnosis	Conventional MRI: standard first examination, including pre- and postcontrast T1-weighted, T2-weighted, and FLAIR imaging [77]. Multiparametric MRI: perfusion and diffusion imaging can contribute to the differential diagnosis [44,45,49].	Can help differentiate neoplastic from non-neoplastic lesions [4,76]	Histomolecular diagnosis is required [4,76]. Note: 20–30% of IDH-mutant CNS WHO grade 2 gliomas show no amino acid uptake.
Grading and molecular prediction	Conventional MRI: contrast enhancement is absent in some high-grade gliomas [3]. Multiparametric MRI: ASL perfusion, ADC, and DKI parameters differ between lower- and higher-grade gliomas [46,47,51].	Can identify the most malignant components, especially in non-enhancing gliomas; dynamic PET parameters correlate with IDH status [4]	Tissue-based histomolecular analysis is required.
Biopsy targeting	Conventional MRI: biopsy is usually directed to contrast-enhancing regions, which may underestimate the tumor grade [3,68]. Multiparametric MRI: MRS may help target the most aggressive tumor region [83].	Areas with the highest uptake are recommended for biopsy guidance [76]	Histomolecular diagnosis is required [4].
Surgical planning	Conventional MRI: basis for neuronavigation; postoperative residual contrast-enhancing and non-enhancing tumor volumes define the prognostic RANO resect classes [23]. Multiparametric MRI: DTI tractography maps eloquent white matter pathways [50].	Uptake may extend beyond contrast enhancement and modify the resection target; residual postoperative uptake has been associated with shorter survival in retrospective studies [4]	Discuss in a multidisciplinary setting and balance the extent of resection against the risk of neurological deficit.
Radiotherapy planning	Conventional MRI: contrast-enhanced T1 and FLAIR MRI are the standard for target volume definition [4,81]. Multiparametric MRI: perfusion- and diffusion-guided dose escalation is under investigation [84].	Can complement MRI in defining the biological tumor volume [4,80,81]	The MRI-based target (resection cavity plus residual enhancing tumor) remains the reference; FLAIR or PET abnormalities are added only when judged to represent tumor, and short-term follow-up PET before irradiation can be considered if reactive postoperative uptake is suspected [81,82].
Recurrence vs. treatment-related change	Conventional MRI: assessment according to RANO 2.0, with confirmatory follow-up MRI if progression is suspected within 12 weeks after radiotherapy [79]. Multiparametric MRI: DSC perfusion [43,78] and ADC [48] can help, although RANO 2.0 deems these techniques as requiring further validation [79].	Can help when MRI findings are equivocal [4,33,34]	Short-term follow-up imaging [75,79]
Response under antiangiogenic or immune therapy	Conventional MRI: assessment according to RANO 2.0 [79]. Multiparametric MRI: diffusion and perfusion MRI may help distinguish progression from pseudoprogression but require further validation [79].	Can help identify pseudoresponse under bevacizumab [4,76]; PET RANO 1.0 has been proposed for clinical trials [75]	If clinically stable, treatment may be continued under close observation, with repeat imaging, e.g., after one to two treatment cycles [75,79]. For clinically unstable patients: consider multidisciplinary discussion and, where feasible, tissue confirmation.

Because MRI is widely available and amino acid PET is not, MRI is generally performed first, and PET is added when a specific question remains unresolved and PET is accessible. This table summarizes the cited guidelines and studies and is not a validated clinical algorithm. ADC: apparent diffusion coefficient; ASL: arterial spin labeling; DKI: diffusion kurtosis imaging; DSC: dynamic susceptibility contrast; DTI: diffusion tensor imaging; FLAIR: fluid-attenuated inversion recovery; MRS: MR spectroscopy; RANO: Response Assessment in Neuro-Oncology.

## Data Availability

No new data were created or analyzed in this study. Data sharing is not applicable to this article.

## Abbreviations

The following abbreviations are used in this manuscript:

[^18^F]FET	O-(2-[^18^F]fluoroethyl)-L-tyrosine
[^18^F]FDOPA	3,4-dihydroxy-6-[^18^F]fluoro-L-phenylalanine
[^11^C]MET	L-[methyl-^11^C]methionine
[^18^F]FACBC	anti-1-amino-3-[^18^F]fluorocyclobutane-1-carboxylic acid ([^18^F]fluciclovine)
[^18^F]FDG	2-deoxy-2-[^18^F]fluoro-D-glucose
5-ALA	5-aminolevulinic acid
ADC	apparent diffusion coefficient
APTw-CEST	amide proton transfer-weighted chemical exchange saturation transfer
ASL	arterial spin labeling
AUC	area under the curve
Cho	choline
CI	confidence interval
CNS	central nervous system
CT	computed tomography
DCE	dynamic contrast-enhanced
DKI	diffusion kurtosis imaging
DSC	dynamic susceptibility contrast
DTI	diffusion tensor imaging
DWI	diffusion-weighted imaging
EANM	European Association of Nuclear Medicine
EANO	European Association of Neuro-Oncology
ESTRO	European Society for Radiotherapy and Oncology
FAPI	fibroblast activation protein inhibitor
FLAIR	fluid-attenuated inversion recovery
GTV	gross tumor volume
HR	hazard ratio
IDH	isocitrate dehydrogenase
IVIM	intravoxel incoherent motion
MGMT	O^6^-methylguanine-DNA methyltransferase
MR	magnetic resonance
MRI	magnetic resonance imaging
MRS	magnetic resonance spectroscopy
NAA	N-acetylaspartate
nCBV	normalized cerebral blood volume
NOS	not otherwise specified
PET	positron emission tomography
PSMA	prostate-specific membrane antigen
PWI	perfusion-weighted imaging
RANO	Response Assessment in Neuro-Oncology
rCBV	relative cerebral blood volume
SNMMI	Society of Nuclear Medicine and Molecular Imaging
SUVmax	maximum standardized uptake value
TBR	tumor-to-background ratio
TBRmax	maximum tumor-to-background ratio
TBRmean	mean tumor-to-background ratio
TERT	telomerase reverse transcriptase
WHO	World Health Organization
